# Apocynin and Nox2 regulate NF-κB by modifying thioredoxin-1 redox-state

**DOI:** 10.1038/srep34581

**Published:** 2016-10-04

**Authors:** Silvia Cellone Trevelin, Célio Xavier dos Santos, Raphael Gomes Ferreira, Larissa de Sá Lima, Rangel Leal Silva, Cristoforo Scavone, Rui Curi, José Carlos Alves-Filho, Thiago Mattar Cunha, Pérsio Roxo-Júnior, Maria-Célia Cervi, Francisco Rafael Martins Laurindo, John Stephen Hothersall, Andrew M. Cobb, Min Zhang, Aleksandar Ivetic, Ajay M. Shah, Lucia Rossetti Lopes, Fernando Queiroz Cunha

**Affiliations:** 1 Department of Pharmacology, Ribeirao Preto Medical School, University of Sao Paulo, Ribeirão Preto, Brazil; 2 Department of Pharmacology, Institute of Biomedical Sciences, University of Sao Paulo, São Paulo, Brazil; 3 King’s College London, British Heart Foundation Centre, Cardiovascular Division, London, United Kingdom; 4 Department of Biophysics and Physiology, Institute of Biomedical Sciences, University of Sao Paulo, São Paulo, Brazil; 5 Department of Pediatrics, Ribeirao Preto Medical School, University of Sao Paulo, Ribeirão Preto, Brazil; 6 Heart Institute, School of Medicine, University of Sao Paulo, São Paulo, Brazil

## Abstract

The reactive-oxygen-species-(ROS)-generating-enzyme Nox2 is essential for leukocyte anti-microbial activity. However its role in cellular redox homeostasis and, consequently, in modulating intracellular signaling pathways remains unclear. Herein, we show Nox2 activation favors thioredoxin-1 (TRX-1)/p40phox interaction, which leads to exclusion of TRX-1 from the nucleus. In contrast, the genetic deficiency of Nox2 or its pharmacological inhibition with apocynin (APO) results in reductive stress after lipopolysaccharide-(LPS)-cell stimulation, which causes nuclear accumulation of TRX-1 and enhanced transcription of inflammatory mediators through nuclear-factor-(NF)-κB. The NF-κB overactivation is prevented by TRX-1 oxidation using inhibitors of thioredoxin reductase-1 (TrxR-1). The Nox2/TRX-1/NF-κB intracellular signaling pathway is involved in the pathophysiology of chronic granulomatous disease (CGD) and sepsis. In fact, TrxR-1 inhibition prevents nuclear accumulation of TRX-1 and LPS-stimulated hyperproduction of tumor-necrosis-factor-(TNF)-α by monocytes and neutrophils purified from blood of CGD patients, who have deficient Nox2 activity. TrxR-1 inhibitors, either lanthanum chloride (LaCl_3_) or auranofin (AUR), also increase survival rates of mice undergoing cecal-ligation-and-puncture-(CLP). Therefore, our results identify a hitherto unrecognized Nox2-mediated intracellular signaling pathway that contributes to hyperinflammation in CGD and in septic patients. Additionally, we suggest that TrxR-1 inhibitors could be potential drugs to treat patients with sepsis, particularly in those with CGD.

Reactive oxygen species (ROS) generated by Nox2 play a vital role in the killing of phagocytized microorganisms. Actually, patients with deficient Nox2 activity due to mutations in oxidase subunits suffer from chronic granulomatous disease (CGD), a condition in which there is increased susceptibility to bacterial and fungal infections^1^. In addition to the key role of Nox2 in the control of infection, there is evidence that Nox2 could limit inflammatory response. For instance, Nox2-deficient mice have higher neutrophil recruitment in a model of thioglycollate-induced peritonitis^2^ or β-glucan ear injection^3^ and increased release of inflammatory mediators in a model of intraperitoneal zymosan challenge^4^. Acute lung injury was also aggravated in mice deficient in p47 phagocyte oxidase (p47phox), a Nox2 subunit^5^. Corroborating the animal models, monocytes from CGD patients exhibit increased lipopolysaccharide-(LPS)-stimulated production of several pro-inflammatory cytokines, such as CCL2, CXCL2, TNF-α and interleukine-[IL]-1β^6^. Furthermore, CGD patients develop aseptic granulomas in several organs, but the underlying molecular mechanism is poorly understood^1^.

Nox2 activation involves the assembly of a multi-subunit complex in which cytosolic p47phox, p40phox and p67phox subunits translocate to a membrane-bound heterodimer comprising the Nox2 catalytic subunit (also known as gp91phox) and a p22phox subunit. The activated enzyme catalyzes electron transfer from nicotinamide adenine dinucleotide phosphate hydrogenated (NADPH) to molecular oxygen, thereby generating superoxide and subsequently other ROS^7^.

Enzymatically derived oligophenols from the peroxidase-catalysed oxidation of apocynin (APO) can be effective inhibitors of Nox2^8^. A previous study demonstrated that APO-trimmer, generate by myeloperoxidase from phagocytes, oxidizes the cysteine residue 196 (Cys196) of p47phox, which impairs its assembly with p22phox^9^.

Although there are no inhibitors of Nox2 directly targeting p40phox, it is a key subunit for Nox2 activation. Neutrophils deficient of p40phox have 85% reduced ROS generation induced by *S. aureus* and IgG-latex beads. In fact, after the cell stimulation, p40phox and p47phox together interact with p67phox and scaffold this subunit on the cell membrane (through phox homology domain [PX]) close to gp91phox^10^. In addition to the regulatory role of p40phox in Nox2 activation, this subunit was also identified as a thioredoxin-1 (TRX-1) binding protein in a yeast two-hybrid-system screen^11^. However, the consequences of this interaction in intracellular signaling pathways have not being investigated.

TRX-1 is a 12 KDa-protein responsible for cell redox homeostasis. It contains an active disulfide/dithiol within the conserved sequence Cys-Gly-Pro-Cys, which allows TRX-1 to operate as a reducing system in association with the selenoprotein thioredoxin reductase-1 (TrxR-1) and NADPH^12^. Inside the nucleus, TRX-1 in its reduced state has the ability to potentiate the binding of transcription factors to DNA^12^.

Nuclear factor-(NF)-κB is one of the systems regulated by TRX-1^13^. The NF-κB subunits p50 and p65 are normally sequestered in the cytoplasm through their tight association with IκB-α protein. After stimuli, for instance the activation of the toll-like-receptor 4 (TLR4) by LPS, IκB-α phosphorylation leads to its proteasomal-degradation and allows the NF-κB subunits to translocate to the nucleus. In the nucleus, p50 and p65 undergo several post-translational modifications, including the reduction of cysteine residues by TRX-1, which leads to their binding to DNA and transcription of several proteins, such as inflammatory mediators and TLRs^12^,^13^,^14^.

In this study, we investigated the interaction between TRX-1 and p40phox after Nox2 activation, and evaluated how changes in TRX-1 redox state induced by Nox2 or APO treatment regulate NF-κB in the settings of CGD and sepsis.

## Results

### The activated Nox2 complex associates with TRX-1 through p40phox

**Figure 1:**
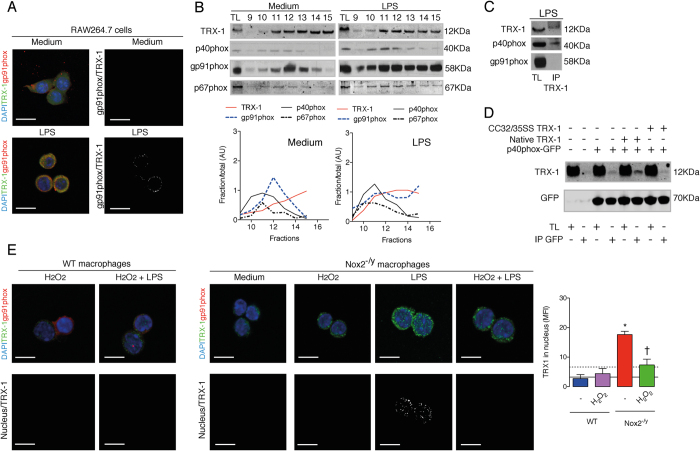
Nox2 activation facilitates thioredoxin-1 (TRX-1) and p40phox interaction and prevents nuclear accumulation of TRX-1. (**A**) TRX-1 (green) and Nox2/gp91phox (red) in RAW264.7 cells stimulated with LPS (10 ng/ml, 30 minutes). Nuclear material was stained with DAPI (blue). Images were obtained by confocal microscopy (63X objective; 5X magnification) and represent three independent experiments realized in triplet. Colocalization is highligted in white/black image. Scale bars, 7.5 μm. (**B**) Sucrose gradient cell fractionation and densitometry. Immunoblots (IB) were performed for TRX-1, p40phox, gp91phox and p67phox in fractions 9 to 15. AU: arbitrary units. (**C**) Immunoprecipitation (IP) was performed with anti-TRX-1 antibody (Ab) in total cell lysates and IB for p40phox, gp91phox and TRX-1. (**D**) HEK293T cells were transfected with p40phox-GFP and either native TRX-1 or mutated CC32/35SS TRX-1. IP was performed with anti-GFP Ab and IB for GFP and TRX-1. Blots represent three independent experiments. TL: total cell lysate. (**E**) Macrophages harvested from WT and Nox2^−/y^ mice were stimulated with LPS. Some cells were treated with hydrogen peroxide (H_2_O_2_, 10 μM). Scale bars, 7.5 μm. The results are expressed as the means of fluorescence intensity (MFI) ±standard error of the mean (SEM) obtained by analyzing 15 nucleus-TRX-1 colocalizations (white) *per* group. Black continuous and dashed lines respectively indicate the average values obtained by analysing WT and Nox2^−/y^ cells in medium only. Full-lengh blots are showed in Supplementary Figs 10 and 11. *P < 0.05 as compared to WT cells incubated with LPS; ^†^*P* < 0.05 as compared to Nox2^−/y^ cells incubated with LPS.

To assess the relationship between Nox2 and TRX-1, the intracellular localization of these proteins was firstly evaluated in leukocytes by using confocal microscopy. LPS stimuli induced the colocalization of gp91phox (Nox2) and TRX-1 outside the nucleus both in RAW 264.7 cells and mouse peritoneal macrophages (Fig. 1A and Supplementary Fig. 1A). Since p40phox and p67phox associate with gp91phox in a protein complex^7^, RAW264.7 cell lysates were subjected to sucrose gradient centrifugation to generate sequential fractions containing TRX-1 and these Nox2 subunits. TRX-1 was recovered predominantly in gels containing fractions 9 and higher (Supplementary Fig. 1B), which were then analyzed for the presence of p40phox, gp91phox (the mouse fully glycosylated form runs with an apparent molecular weight of 58 KDa) and p67phox. Under baseline conditions, there was a modest overlap between TRX-1 and Nox2 subunits whereas lysates of LPS-stimulated cells showed a substantial proportion of TRX-1, gp91phox, p40phox and p67phox recovered in the same sucrose gradient fractions, consistent with the notion that protein association mainly occurs when Nox2 is activated (Fig. 1B). As controls, albumin and cytochrome c, which have comparable molecular weights to gp91phox and TRX-1 respectively, were found in distinctly separate fractions (Supplementary Fig. 1C). When cell lysates were treated with N-ethylmaleimide (NEM, the thiol alkylating agent that blocks all cysteine residues of proteins^15^), Nox2 (gp91phox) and TRX-1 were no longer recovered in the same fractions (Supplementary Fig. 1D), indicating a redox dependent interaction. In order to identify which Nox2 subunit directly interacts with TRX-1 after LPS stimuli, immunoprecipitation assays were performed in RAW cells lysates. Anti-TRX-1 antibody failed to recover gp91phox (Fig. 1C) and neither TRX-1 was recovered in anti-myc immunoprecipitates of HEK293T cells transfected with Nox2-(gp91phox)-myc and native TRX-1 (Supplementary Fig. 1E). However, p40phox was co-immunoprecipitated with TRX-1 in RAW264.7 cell lysates (Fig. 1C). Furthermore, native TRX-1 could be co-precipitated with p40phox in anti-green-fluorescent-protein-(GFP) immunoprecipitates of HEK293T cells transfected with p40phox-GFP (Fig. 1D). Reinforcing the results obtained from sucrose gradient fractionation of NEM-incubated samples, a TRX-1 mutant in that serine residues replaced Cys32 and Cys35 did not interact with p40phox (Fig. 1D). These combined results suggest that thiol redox-regulation of active cysteine residues of TRX-1 by Nox2 is essential for its interaction with p40phox.

The localization of TRX-1 in cells lacking functional Nox2 was next analyzed. APO was used as an inhibitor of Nox2 in RAW264.7 cells. In fact, APO significantly impaired the colocalization of p47phox and Nox2 (gp91phox) after LPS-stimulation, and almost abolished the ROS generation stimulated by opsonized zymosan (Supplementary Fig. 2A,B). The colocalization of Nox2 (gp91phox) and TRX-1 was not observed in LPS-stimulated RAW264.7 cells treated with APO and macrophages from Nox2^−/y^ mice (chromosome X-linked genetic deficiency of Nox2), and instead there was a nuclear accumulation of TRX-1 (Fig. 1E and Supplementary Fig. 1F). Moreover, the addition of H_2_O_2_ was sufficient to prevent this last response in macrophages from Nox2^−/y^ mice (Fig. 1E).

Therefore, after LPS stimulation, TRX-1 interacts with the activated Nox2 complex through p40phox, thereby preventing its nuclear accumulation.

### APO modifies the TRX-1 redox-state and increases NF-κB binding to DNA through Nox2 inhibition

**Figure 2:**
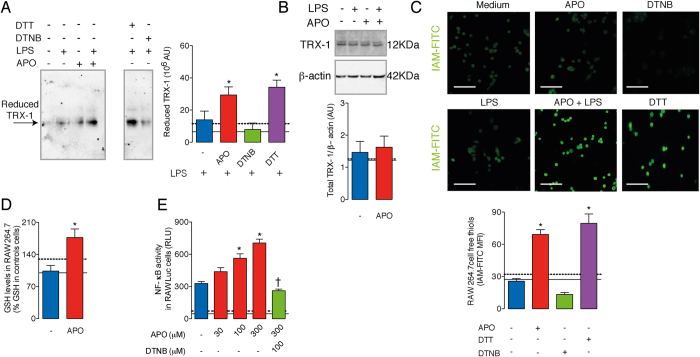
Inhibition of Nox2 by APO results in TRX-1 reduction and in a reductive stress after LPS stimuli. RAW264.7 cells were incubated with APO (300 μM, one hour) and stimulated with LPS (10 ng/ml, 30 minutes). Some cells were incubated with 5,5′-dithiobis-2-nitrobenzoic acid (DTNB, 100 μM) or dithiothreitol (DTT, 300 μM). Immunoblotting was performed under native (**A**) or reducing condition (**B**). Densitometry of three independent experiments. The results are expressed as the means ± SEM. AU: arbitrary units. (**C**) Levels of free thiols were determined by using IAM-FITC (green). The results are expressed as the means of fluorescence intensity (MFI) ± SEM obtained by analyzing 15–20 cells/group. Scale bars, 50 μm. (**D**) Levels of reduced glutathione (GSH) in total cell lysates. (**E**) RAW264.7-Luc cells were incubated with APO and then stimulated with LPS (10 ng/ml, four hours). Some cells were incubated with DTNB 30 minutes before the addition of APO. RLU: relative lumen units. Black continuous and dashed lines respectively indicate the average values obtained by analysing cells incubated in medium or after APO-treatment only. The results are expressed as the means ± SEM (n = 6/group, samples incubated with LPS; n = 3/group, samples without LPS). Full-lengh blots of the (**B**) are showed in Supplementary Fig. 12A. *P < 0.05 as compared to cells incubated with LPS; ^†^*P* < 0.05 as compared to LPS-stimulated cells pre-incubated with APO.

Mammalian TRX-1 has three non-active cysteine residues (Cys62, Cys69, and Cys73) in addition to two redox-active cysteine residues (Cys32-Cys35). Therefore, mouse TRX-1 can assume several oxidation states that are poorly visualized in a redox immunoblotting analysis, whereas TRX-1 in a fully reduced state is clearly resolved^16^,^17^. After alkylation (blocking) of thiols with iodoacetic acid and then analysis on native polyacrylamide gels, lysates of RAW264.7 cells pre-treated with APO and stimulated with LPS showed significantly higher levels of reduced TRX-1 as compared to lysates of cells incubated with LPS only (Fig. 2A). Lysates from DTT-(dithiothreitol, reducing agent)-treated cells were used as positive controls for reduced TRX-1^15^. There was no difference in total TRX-1 protein levels evaluated by conventional immunoblotting methods (Fig. 2B).

Because TRX-1 redox-state was modified by APO treatment and this reductase is recognized as an important modulator of cell redox homeostasis^18^, we reasoned that Nox2 inhibition or its genetic deficiency would result in a more reduced intracellular environment and hence higher levels of free thiol groups. Iodoacetamide conjugated to isothiocyanate fluorescein (IAM-FITC) was used as an alkylating agent that interacts with free thiol groups^15^. Cells incubated with DTT and 5,5′-dithiobis-2-nitrobenzoic acid (DTNB, oxidizing agent) were used as controls. LPS-stimulated RAW264.7 cells treated with APO showed a substantial increase in IAM-FITC fluorescence as assessed by confocal microscopy and flow cytometry (Fig. 2C and Supplementary Fig. 3A). Similar results were observed in macrophages from Nox2^−/y^ mice stimulated with LPS (Supplementary Fig. 3B). Of note, APO plus LPS-treated Nox2^−/y^ macrophages showed IAM-FITC staining comparable to that presented by LPS-treated Nox2^−/y^ cells (Supplementary Fig. 3B), indicating that APO *per se* does not change cellular redox state independently of Nox2 inhibition. Consistent with the IAM-FITC assay, reduced glutathione (GSH) levels were increased in the LPS-stimulated RAW264.7 cells pre-treated with APO as compared to cells under LPS stimuli only (Fig. 2D).

Disturbances in NF-κB activation were further investigated as consequences of the lack of cell redox homeostasis. In fact, LPS-induced NF-κB activity was inhibited by DTNB oxidizing agent and enhanced by DTT reducing agent in a luciferase reporter assay using RAW264.7-Luc cells (Supplementary Fig. 3C), which is in accordance with a previous study^19^. Similarly to the DTT effect on NF-κB, the reductive stress^20^ resulting from APO-treatment potentiated LPS-induced NF-κB activation in a concentration-dependent manner (Fig. 2E). Moreover, the effect of the highest concentration of APO was prevented by cellular oxidation with DTNB (Fig. 2E). Importantly, none of the agents employed in this study affected cell viability (Supplementary Fig. 3D).

**Figure 3:**
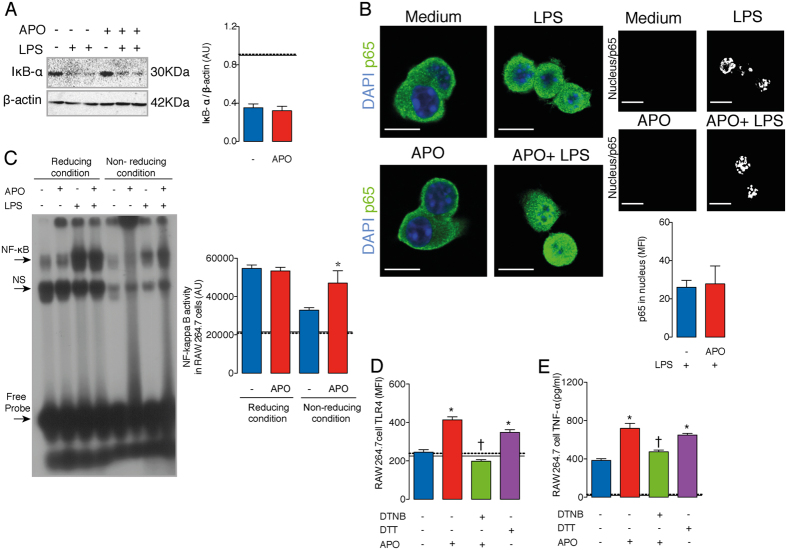
Inhibition of Nox2 by APO increases LPS-mediated NF-κB binding to DNA. RAW264.7 cells were incubated with APO (300 μM, one hour) and then stimulated with LPS (10 ng/ml, 30 minutes). (**A**) Degradation of IκB-α, 15 minutes after LPS. Densitometry of three independent experiments. AU: arbitrary units. (**B**) Translocation of p65 (green) to nucleus (blue), 30 minutes after LPS. Images were acquired by confocal microscopy (63X objective; 5X magnification). Scale bars, 7.5 μm. The results are expressed as the means of fluorescence intensity (MIF) ±SEM obtained by analysing 15 nucleus-TRX-1 colocalizations (white) *per* group. (**C**) Eletromobility shift assay (EMSA) under reducing (plus DTT, 1 mM) or non-reducing conditions. NS: non-specific. (**D**) TLR4 expression by flow cytometry, 12 hours after LPS. (**E**) TNF-α levels in culture supernatants (ELISA), 12 hours after LPS. The results are expressed as the means ± SEM (n = 6/group, samples incubated with LPS; n = 3/group, samples without LPS). Black continuous and dashed lines respectively indicate the average values obtained by analysing cells incubated in medium or after APO-treatment only. Full-lengh blots of the (**A**) are showed in Supplementary Fig. 12B. *P < 0.05 as compared to cells incubated with LPS; ^†^*P* < 0.05 as compared to LPS-stimulated cells pre- incubated with APO.

To further define the molecular mechanism through which inhibition of Nox2 enhances NF-κB activation, IκB-α degradation and the nuclear translocation of the p65 NF-κB subunit in LPS-stimulated RAW264.7 cells were monitored. Treatment with APO had no significant effect on IκB-α degradation or p65 translocation (Fig. 3A,B), suggesting that Nox2 is not acting upstream of these steps. However, NF-κB binding to DNA was enhanced by APO in LPS-stimulated cells, as assessed by an electrophoretic mobility shift assay (EMSA) under non-reducing conditions (*i.e.* in the absence of DTT). When EMSA was performed under reducing conditions, NF-κB binding to DNA was enhanced independently of Nox2 inhibition (Fig. 3C). A specific non-radioactive probe was used as a control in the EMSA assay; and anti-p65 and anti-p50 NF-κB antibodies favored the super-shift in nuclear extracts of LPS-stimulated cells (Supplementary Fig. 4). In line with an over activation of NF-κB after Nox2 inhibition, LPS-stimulated RAW264.7 cells pre-incubated with APO had increased TLR4 expression and higher TNF-α production than cells in LPS only. These two responses were prevented by DTNB and mimicked those of DTT (Fig. 3D,E). Corroborating our results obtained with leukocytes treated with APO, neutrophils and macrophages purified from Nox2^−/y^ mice had higher transcription of inflammatory molecules (TNF-α, TLR4) induced by LPS as compared to cells of WT mice (Supplementary Fig. 5A–D). Also consistent with our data, the LPS-induced TLR4 expression in leukocytes from Nox2^−/y^ mice was prevented by incubation with an NF-κB inhibitor, BAY 11-7082 (inhibits IκB-α phosphorylation^21^, [Supplementary Fig. 5E,F]).

**Figure 4:**
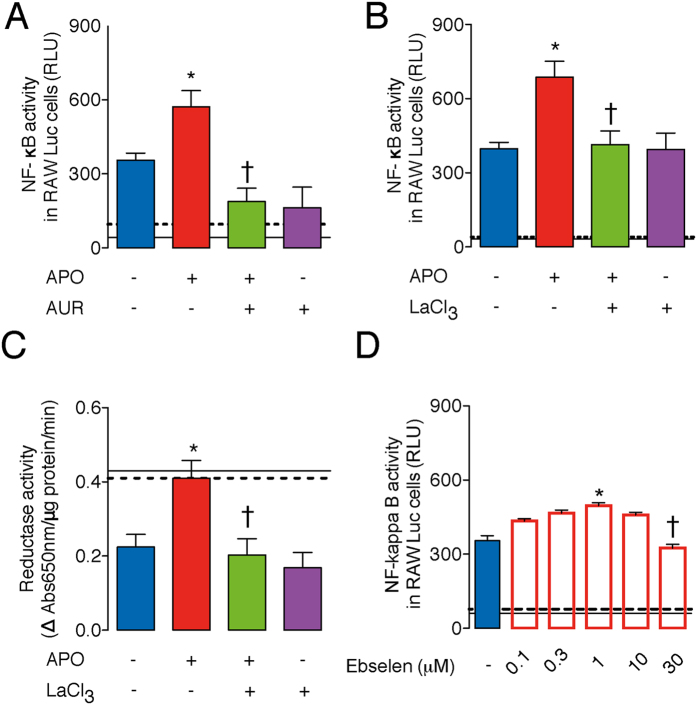
TrxR-1 inhibition prevents the LPS-mediated NF-κB over activation induced by APO. RAW264.7-Luc cells were incubated with APO (300 μM, one hour) and then stimulated with LPS (10 ng/ml, four hours). (**A**,**B**) Cells incubated with the TrxR-1 inhibitors, auranofin (AUR, 1 μM- [A]) or lanthanum chloride (LaCl_3_, 1 μM- [B]), for 30 minutes before APO. RLU: relative lumen units. (**C**) Reductase activity of cell lysates 30 minutes after LPS. (**D**) Cells were incubated with ebselen one hour before LPS. The results are expressed as the means ± SEM (n = 6/group, samples incubated with LPS; n = 3/group, samples without LPS). Black continuous and dashed lines indicate the average values obtained by analysing cells incubated in medium or APO-treatment (or ebselen-treatment 30 μM in (**D**), respectively. *P < 0.05 as compared to cells incubated with LPS; ^†^*P* < 0.05 as compared to LPS-stimulated cells pre- incubated with APO.

Since thioredoxin reductase-1 (TrxR-1) is the enzyme responsible for reduction of TRX-1 in the cytoplasm^22^,^23^, we further investigated whether TrxR-1 inhibition, and the consequent redox shift of TRX-1 from its reduced to oxidized state, could prevent the APO-induced NF-κB over activation. In fact, LPS-stimulated RAW264.7-Luc cells treated with APO and pre-incubated with auranofin (AUR, which blocks the selenocysteine 496 of TrxR-1^22^) or lanthanum chloride (LaCl_3_, which inhibits the NADPH interaction site of TrxR-1^23^), showed a markedly lower NF-κB activation than cells treated with APO alone (Fig. 4A,B). Moreover, lysates from APO plus LPS-treated cells showed higher TRX-1-mediated reductase activity than lysates from cells in LPS only, which was prevented by LaCl_3_ (Fig. 4C). This latter result confirmed the APO induction of the reductive stress after LPS incubation through augmentation of reduced TRX-1 levels, as well as the ability of LaCl_3_ to prevent this altered cellular redox-status.

Ebselen is an organoselenium compound previously associated with inhibition of Nox2 by preventing the assembly of p47phox and p22phox^24^. However, this drug is also able to competitively antagonize TrxR-1 or even directly oxidizes TRX-1^25^. At lower concentrations (0.1 to 1 μM), ebselen increased NF-κB activation, similar to APO, which could be associated with inhibition of Nox2 (Fig. 4D). In contrast, at higher concentrations (10 to 30 μM), ebselen did not change or inhibit NF-κB activation, corresponding to antagonism of TrxR-1 and/or oxidation of TRX-1 (Fig. 4D). Importantly, ebselen at 30 μM decreased TRX-1-mediated reductase activity of RAW264.7 cells with or without LPS stimuli (Supplementary Fig. 6).

### TrxR-1 inhibition prevents the aberrant TNF-α production by LPS-stimulated leukocytes from CGD patients

**Figure 5:**
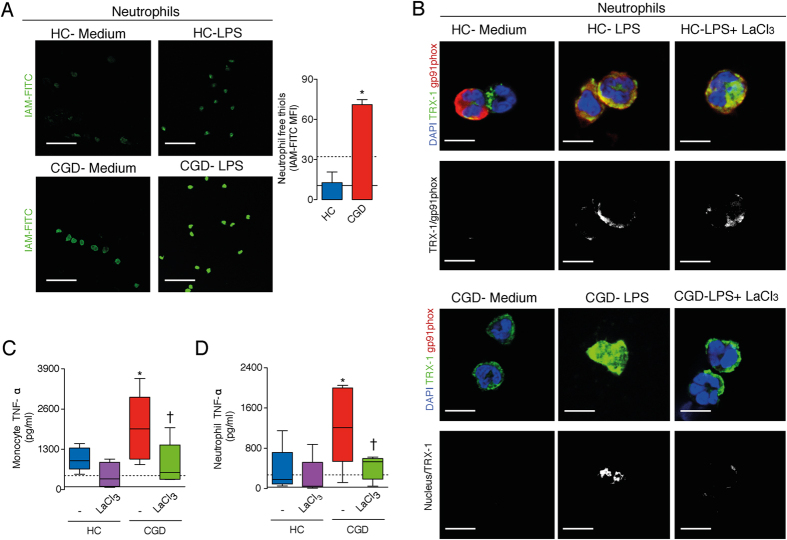
TrxR-1 inhibition prevents aberrant TNF-α production by leukocytes from chronic granulomatous disease patients (CGD) stimulated with LPS. (**A**–**C**) Neutrophils from healthy controls (HC, n = 5) or CGD patients (n = 5) were stimulated with LPS (10 ng/ml). (**A**) Staining with IAM-FITC (green), 30 minutes after LPS. Images were obtained by confocal microscopy (63X objective). Scale bars, 50 μm. The results are expressed as the means fluorescence intensity (MIF) ± SEM obtained by analyzing 15 cells/subject. (**B**) Colocalization (white) of TRX-1 (green) and Nox2 (gp91phox, red) in HC cells and nuclear accumulation of TRX-1 (white) in cells of CGD cells, 30 minutes after LPS. Nuclear material was stained with DAPI (blue). Images were obtained by confocal microscopy (63X objective; 5X magnification). Scale bars, 7.5 μm. (**C**,**D**) TNF-α levels in cultures supernatant (ELISA), 12 hours after LPS. Some cells were treated with lanthanum chloride (LaCl_3_, 1 μM) 30 minutes before LPS. Box plots show median, interquartile range, sample minimum and maximum. Black continuous and dashed lines indicate the average values obtained by analysing, respectively, HC and CGD cells in medium only. *P < 0.05 as compared to HC cells stimulated with LPS; ^†^*P* < 0.05 as compared to CGD cells stimulated with LPS.

The relevance of the Nox2/TRX-1/NF-κB intracellular signaling pathway was investigated in humans using leukocytes from healthy controls (HC) and patients with CGD (Supplementary Table S1). LPS-stimulated neutrophils and monocytes from CGD patients showed a substantially higher thiol reduced state than those isolated from HC (Fig. 5A and Supplementary Fig. 7). As in murine cells, LPS stimulation induced the colocalization of Nox2 and TRX-1 in neutrophils of HC, whilst there was a nuclear accumulation of TRX-1 in cells of CGD patients (Fig. 5B). The higher levels of TRX-1 in the nucleus were in line with an enhanced LPS-stimulated production of TNF-α by cells from CGD patients as compared to those from HC (Fig. 5C,D). Furthermore, treatment of neutrophils of CGD individuals with LaCl_3_ (TrxR-1 inhibitor) prevented the nuclear accumulation of TRX-1, and, as a consequence, decreased the LPS-stimulated production of TNF-α (Fig. 5B,D). LaCl_3_ also prevented the aberrant TNF-α production by monocytes from CGD subjects (Fig. 5C).

Altogether these results demonstrate that, after LPS stimuli, a deficient Nox2 activity results in increased levels of reduced TRX-1 into the nucleus leading to a NF-κB over activation.

### Nox2 limits the severity of sepsis through TRX-1/NF-κB signaling pathway

**Figure 6:**
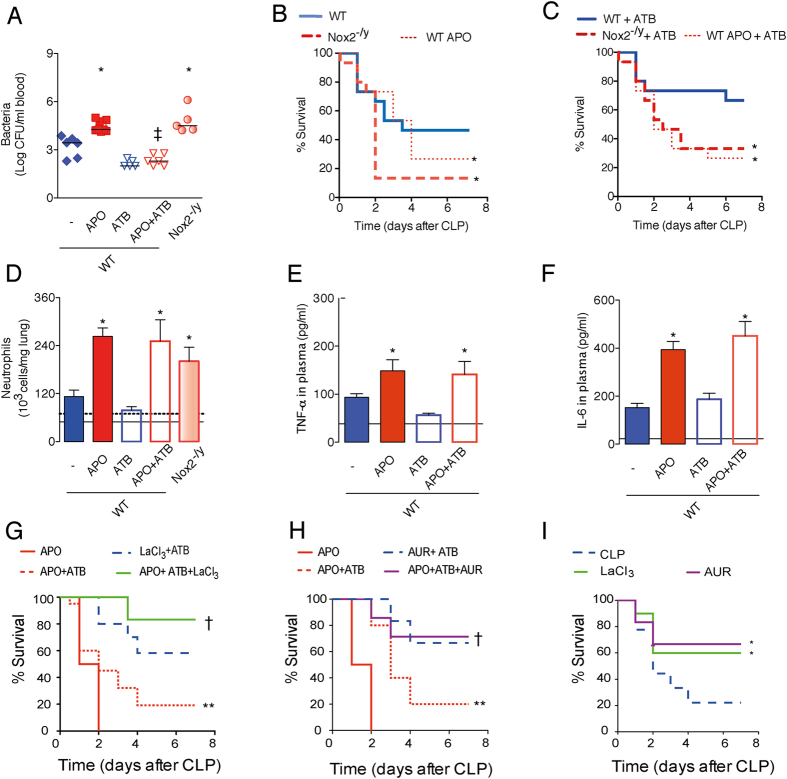
TrxR-1 inhibition enhances sepsis survival of mice treated with APO. Wild type (WT) mice subcutaneously treated with APO (200 mg/kg, 30 minutes before surgery) and Nox2^−/y^ mice were submitted to cecal ligation and puncture (CLP). Some mice were intraperitonously treated with antibiotic (ATB, ertapenem sodium, 30 mg/kg), six hours after surgery and 12 hourly thereafter. (**A**) Bacterial load in blood, 12 hours after CLP. The graph represents the individual logarithmic values of CFU around median (n = 5–7/group). (**B**,**C**) Survival rates (n = 15/group). (**D**–**F**) Number of neutrophils trapped in lungs, levels of TNF-α and IL-6 in plasma, six hours after ATB treatment and 12 hours after CLP. The results are expressed as the means ± SEM (n = 5–7/group). Black continuous and dashed lines in bar graphs indicate the average values obtained by analysing WT and Nox2^−/y^ sham-operated mice, respectively. (**G**–**I**) Mice were treated with LaCl_3_ (10 mg/kg, subcutaneously), which was administered six hours after surgery and 12 hourly thereafter; or mice were treated with auranofin (AUR, 2 mg/kg, subcutaneously), which was administered six hours after surgery and 24 hourly thereafter. Survival rates (n = 7–10/group; n = 7, AUR and/or APO treated mice; n = 10, LaCL_3_ and or APO treated mice). *P < 0.05 as compared to WT-CLP mice; ^†^*P* < 0.05 as compared to APO-CLP mice under antibiotic therapy; ^‡^*P* < 0.05 as compared to APO-CLP mice without ATB treatment; **P < 0.05 as compared to LaCl_3_ -CLP or AUR -CLP mice, both under antibiotic therapy.

Sepsis is the major cause of mortality in critically ill patients. During sepsis, invading pathogens trigger a systemic inflammatory response syndrome (SIRS), which requires the activation of NF-κB by microbial products, such as LPS^26^,^27^,^28^. It was reasoned that the Nox2/TRX-1/NF-κB intracellular signaling pathway triggered by LPS is implicated in sepsis pathophysiology. ROS-production was evaluated in blood and peritoneal leukocytes from mice deficient in Nox2 (Nox2^−/y^) and those wild type (WT) treated with APO. All these mice were submitted to a mouse cecal ligation and puncture (CLP) model of sepsis. Sepsis induced an increased ROS-generation by leukocytes systemically and at the site of infection, which was severely impaired in both Nox2^−/y^ mice and APO-treated mice (Supplementary Fig. 8A,B). APO treatment or the genetic deficiency of Nox2 increased bacterial load in blood (Fig. 6A) and reduced survival rates after CLP in comparison with WT septic mice (Fig. 6B). The fact that either Nox2 deficient or APO- treated mice presented similar number of leukocytes/neutrophils into the infectious foci (Supplementary Fig. 8C,D), as compared to control mice, reinforces that the increased bacterial load is a consequence of an impaired leukocyte microbicidal ability (Supplementary Fig. 8E). Interestingly, both Nox2^−/y^ and APO-treated mice submitted to CLP had higher numbers of neutrophils trapped in the lungs as compared to controls (Fig. 6D), which is a hallmark of an overwhelmed SIRS^28^. Next, the role of Nox2 was determined when the mice were under antibiotic therapy (analogous to the clinical management of sepsis^26^). Mice received a microbicidal antibiotic (ATB, ertapenem sodium, 30 mg/kg), six hours after CLP and 12 hourly thereafter. Although antibiotic treatment reduced bacteremia in APO-treated mice to a level comparable to that in control mice (Fig. 6A), survival rates remained significantly lower in mice with deficient Nox2 activity (Fig. 6C). Consistent with an increased SIRS independent of bacterial killing but related to an over activation of NF-κB, APO-treated mice under antibiotic therapy had aggravated neutrophil trapping in the lungs, and increased plasma levels of TNF-α, IL-6 (Fig. 6D–F), aspartate aminotransferase (AST) and blood urea nitrogen (*i.e.* markers of renal dysfunction and liver injury, respectively- Supplementary Fig. 9A,B). In the setting of sepsis undergoing antibiotic therapy, the inhibition of TrxR-1 by either LaCl_3_ or AUR significantly increased the survival rates of APO-treated mice (Fig. 6G,H). Moreover, both TrxR-1 inhibitors increased the survival rates of WT mice submitted to CLP, even in the absence of antibiotic therapy (Fig. 6I). This strongly implies the Nox2/TRX-1/NF-κB signaling pathway in the pathophysiology of sepsis.

**Figure 7:**
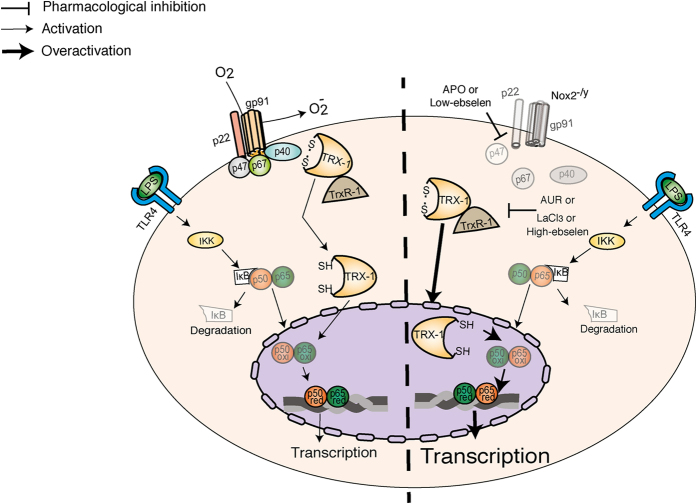
Nox2 regulates NF-κB activation by modifying TRX-1 redox-state. Schematic representation. Left panel: Nox2 assembly favors TRX-1 interaction with p40phox. Oxidized TRX-1 is excluded from the nucleus by p40phox until its reduction by thioredoxin reductase-1 (TrxR-1). The TRX-1 reduction allows its nuclear localization, where it facilitates NF-κB binding to DNA. Right panel: the genetic deficiency (Nox2^−/y^) or pharmacological inhibition of Nox2 (with apocynin, APO; or low-concentration-ebselen) results in reduction of TRX-1 and its nuclear accumulation. In the nucleus, reduced TRX-1 potentiates NF-κB binding to DNA, and consequently, enhances the transcription of inflammatory mediators. TrxR-1 inhibitors, such as lanthanum chloride (LaCl_3_), auranofin (AUR) or high-concentration-ebselen prevent overactivation of NF-κB.

Overall, our results revealed a novel Nox2/TRX-1/NF-κB intracellular signaling pathway (Fig. 7) which contributes to better understanding of the hyperinflammation in CGD patients and also suggests TRX-1 as a novel therapeutic target in sepsis.

## Discussion

It is well established that neutrophil Nox2 is critical for the killing of phagocytized pathogens and its deficiency is associated with increased susceptibility of CGD patients to sepsis and other kinds of infections^1^. Indeed, neutrophils from Nox2^−/y^ or WT pre-incubated with APO showed a significant deficiency in killing, which might be associated to high bacterial load in blood found in Nox2^−/y^ and APO-treated mice submitted to CLP. Nevertheless, even after antibiotic treatment and effective control of infection, deficient Nox2 activity resulted in increased systemic inflammatory response and multi-organ dysfunction. Actually, the Nox2 anti-inflammatory effects were extensively demonstrated in murine models^2^,^3^,^4^,^5^ and in CGD patients^1^,^6^, however the precise intracellular mechanisms mediated by Nox2 have not being elucidated previously. In this study, a Nox2/TRX-1/NF-κB signaling pathway implicated in sepsis and CGD induced-hyperinflammation was identified (Fig. 7).

Although the interaction between the Nox2 subunit p40phox and TRX-1 was previously established^11^, the real consequences of this association in cell redox homeostasis and intracellular signaling have been neglected. Herein, TRX-1/p40phox interaction was observed preferentially after Nox2 assembly. In fact, p40phox is phosphorylated downstream of TLR4 activation by LPS, which allows its assembly with p67phox and Nox2^10^. It was also verified that TRX-1/p40phox interaction depends on the cysteine residues 32 and 35, that encompass the active redox site of TRX-1. In fact, ROS produced by Nox2 followed by LPS stimuli directly modified TRX-1 redox state, which regulated cell redox status. On the other hand, cells with deficient Nox2 activity stimulated with LPS had a reductive stress (increased levels of GSH and other free thiol groups^20^) and increased levels of TRX-1 in the nucleus.

NF-κB is a major transcription factor involved in the synthesis of inflammation-related molecules^14^ and therefore it is a likely target for Nox2-dependent anti-inflammatory effects. We found that the mechanism of increased NF-κB activity in a Nox2-deficient setting was due to a TRX-1 induction of a reductive stress, which was prevented by the oxidizing agent (DTNB). These results are consistent with previous findings that NF-κB binding to DNA is modulated by oxidation/reduction^19^ and that p47phox-deficient mice have enhanced NF-κB binding to DNA in lung extracts after intraperitoneal LPS*-*treatment^5^. Importantly, the higher levels of reduced TRX-1 inducing a reductive stress may not only directly modulate NF-κB activity but also alter the redox state of other cysteine-containing proteins, such as ref. 1, which also enhances NF-κB/DNA binding^5^. Furthermore, oxidation of TRX-1 (through LPS-induced Nox2 activity or treatment with H_2_O_2_ or with TrxR-1 inhibitors) excluded TRX-1 from the nucleus, which was attributed to an increased binding with p40phox. The TRX-1/p40phox/Nox2-dependent mechanism is consistent with the emerging theme of Nox-dependent redox signaling, in which oxidase regulatory subunits (*e.g.* p40phox and p47phox) act as scaffolds that colocalize the activated Nox2 complex with redox-sensitive signaling targets within a cellular microdomain^29^. The involvement of reduced TRX-1 in NF-κB over activation induced by Nox2 deficiency was corroborated by results with LaCl_3_, AUR and high-concentration-ebselen that shifted the redox equilibrium of TRX-1 towards its oxidized state by inhibiting TrxR-1. Indeed, either LaCl_3_ or AUR prevented NF-κB over activation in APO-treated RAW264.7 cells. Furthermore, LaCl_3_ inhibited the exaggerated LPS-induced TNF-α synthesis observed in monocytes and neutrophils of human CGD patients and it also increased the survival rates of APO-treated mice submitted to CLP, providing clear evidence for the *in vivo* efficacy of targeting the Nox2/TRX-1/NF-κB pathway.

ROS-produced by Nox2 controlled inflammation and infection in early sepsis. Therefore, Nox2 is protective. Accordingly, a previous study evaluating septic patients concluded that the severity of the disease was inversely correlated with ROS production by circulating neutrophils^30^. Likewise, a recent meta-analysis involving 2,768 patients observed that the antioxidant, N-acetyl cysteine, did not improve sepsis outcome but was rather associated with cardiovascular instability^31^. Although, we have previously verified that either apocynin treatment or Nox2 deficiency reduces inflammation of the central nervous system, five days after CLP^32^, the beneficial role of Nox2 during sepsis in early phases of the disease cannot be neglected. In the present study we verified the total mortality rates of APO-treated or Nox2^−/−^ mice were higher as compared to WT mice. In fact, the higher systemic inflammation in early phases of sepsis is essential to determine long-term survival^26^. We have also verified APO-treated or Nox2^−/−^ mice present a higher inflammatory response as compared to WT, 12 hours post-CLP, which resulted in higher multi-organ dysfunction and death. Additionally, the transcription of TLR4 and TNF-α was upregulated in neutrophils and peritoneal macrophages under Nox2-deficient conditions, which may explain the increased neutrophil sequestration in the lungs of APO-treated and Nox2^−/y^ mice subjected to CLP^28^.

Recently, some studies have shown an association between Nox2 deficiency and an altered Ca^2+^ influx^33^,^34^,^35^. In this regard, LaCl_3_, in addition to its role inhibiting TrxR-1, is also able to block Ca^2+^ channels^23^ and to impair the binding of LPS to monocytes^36^. Since, it was verified that LaCl_3_ increased survival rates of APO-treated mice submitted to CLP, we cannot exclude the additional protective effect of *in vivo* Ca^2+^ blockade in endothelial cells or platelets, which could prevent vascular dysfunction in sepsis. Nevertheless, the potential of LaCl_3_ to prevent the reductive stress in the setting of deficient Nox2 activity was confirmed through analyses of results obtained from TRX-1-reductase activity assay and the capacity of LaCl_3_ to exclude TRX-1 from the nucleus of CGD neutrophils treated with LPS. These effects of LaCl_3_ were independent of LPS cell binding or the altered Ca^2+^ influx. Moreover, the ability of LaCl_3_ in preventing the APO-induced NF-κB over activation and the CLP-induced mortality were similar to those observed with auranofin (AUR), which is a gold standard TrxR-1 inhibitor devoid of Ca^2+^ related effects^22^.

It was previously demonstrated that intermediates of APO oxidation are able to scavenge the GSH^37^. Therefore, these APO-derived compounds could prevent the reductive stress resultant from the failure of Nox2 activation in the cells under LPS stimulation. However, this last hypothesis was not observed in the present study. Actually, APO-treatment did not prevent the reductive stress (enhanced IAM-FITC staining) of Nox2 deficient macrophages stimulated with LPS. Thus, our results regarding NF-κB over activation after APO-treatment are dependent on Nox2 inhibition and are not due to a direct effect of APO-intermediates on cellular redox status.

Altogether, the current results identify a hitherto unrecognized Nox2-regulated TRX-1 pathway that promotes the control of NF-κB-dependent transcription of inflammatory mediators (Fig. 7). These findings may be especially relevant in CGD patients, and allow us to suggest TrxR-1 inhibitors as novels therapeutic approaches to target hyperinflammation. Furthermore, we have verified that TrxR-1 inhibitors, either LaCl_3_ or AUR, increase sepsis survival. Therefore, they could also be potential drugs to treat septic patients in future.

## Methods

### Animals

Experiments were performed in six-week-old adult male mice of the C57BL/6 strain. Nox2^−/y^ mice were obtained from Jackson Laboratories and bred locally. CLP and sham procedures were performed under intraperitoneal ketamine/xylazine anesthesia as previously described^38^. Animal care, handling and surgical procedures were in accordance with The Ethical Principle in Animal Research adopted by Brazilian College of Animal Experimentation (COBEA). All studies using animals were approved by The Ethical Commission of Ethics in Animal Research (CETEA) of Ribeirao Preto Medical School, University of Sao Paulo, Ribeirão Preto, São Paulo State, Brazil (protocols no 032/2011 and 062/2013).

### Leukocyte purification

Mouse neutrophils were purified from bone marrow (BM) as described previously^38^. Peritoneal macrophages were harvested by instilling 1.5 ml PBS containing 1 mM EDTA into the abdomen and subsequent adherence on acrylic culture plates. Human neutrophils and monocytes were isolated by a standard four-layer Percoll gradient method (45, 54, 63 and 75%). Erythrocytes were depleted in NH_4_Cl buffer (0.14 M).

### Bacterial counts

Peritoneal exudate and blood were collected under sterile conditions, plated on Muller-Hinton agar dishes (Difco Laboratories, USA), and incubated at 37 °C. Colony forming units (CFU) were recorded after 18 hours.

### Immunofluorescence

Cells were fixed with 4% paraformaldehyde and permeabilized with 0.2% Triton-X. Primary antibodies (Abs) were used at 1:100 to 1:200 dilution. Nucleus were stained with 4,6-diamidino-2-phenylindole (DAPI). To determine the levels of thiol free groups, paraformaldehyde-fixed cells were incubated with 0.75 mM 6-iodoacetamide fluorescein (IAM-FITC, Sigma Aldrich, St. Louis, MO) for 30 minutes. DTT (reducing agent) or DTNB (oxidizing agent) were used as controls. Images were acquired by confocal microscopy (TCS SP5 II, Leica Microsystems). Fluorescencense intensity was quantified by using Image J software (Image Processing and Analysis in Java, Mac OS X 10.8 application, Wayne Rasband, National Institutes of Healthy, USA). The colocalization analysis was performed using the plugin *Colocalization highligter* available in the same software.

### NF-κB activity

Experiments were performed in a RAW264.7 cell line containing an NF-κB promoter-luciferase construct (pNF-κB-Luc). Luciferase activities in cell lysates were determined by using the Luciferase 1000 assay system (Promega) in a luminometer (GloMax 20/20 Single tube luminometer, Promega).

### Immunoblotting, sucrose gradient and immunoprecipitation

Immunoblotting was performed using a standard protocol. The redox state of TRX-1 was determined in native gels without additon of 2-β-mercaptoethanol in samples, as described previously^17^. Thiol groups in cell lysates were firstly alkylated with iodoacetic acid (50 mM) in a 6 M guanidine-HCl buffer with 0.5% Triton X-100. For sucrose gradient separation, cell lysates were laid on the top of a four-layer sucrose gradient (10, 20, 40 and 60%). Samples were centrifuged at 35,000 g for 18 hours and fractions were submitted to polyacrilamide gel electrophoresis (SDS-PAGE). A mix of proteins from 12–200 kDa (Gel filtration molecular weight markers, Sigma-Aldrich) was used as control in sucrose grandient centrifugation, with the separated proteins stained with Coomassie Blue. For immunoprecipitation analyses, cell lysates were incubated with anti-TRX-1, protein A/G and agarose immunoprecipitation reagent (Santa Cruz), green fluorescent protein-(GFP)-Trap A (Chromo Tek GmbH, Munich, Germany) or anti- myc immunoprecipitation kit (Sigma-Aldrich, St. Louis, USA) as appropriate. Protein band densitometry was quantified by using Image J software.

### Reductase activity

The insulin reduction assay was used to estimate TRX-1 mediated reductase activity^16^. The precipitation of the insulin β-chain was initiated by adding 1 mM DTT in a 100 μM sodium phosphate buffer (pH 6.5) containing 2 mM ethylenediamine-tetraacetic-acid (EDTA) and insulin (Lanthus^®^, insulin glargine rDNA origen- final concentration of 1 U/ml) to cell lysates. The analysis was followed at 650 nm in a microplate reader coupled to a spectrophotometer (SpectraMax M5, Molecular Devices, Sunnyvale, CA, USA) and corrected by total protein levels determined with Bradford reagent (Sigma-Aldrich).

### EMSA

EMSA was performed as described previously^39^. NF-κB double-stranded consensus oligonucleotide (5′-AGTTGAGGGGACTTTCCCAGGC-3′) was end-labeled with γ-^32^P-ATP and unincorporated nucleotides removed by using a Sephadex G-25 spin column. Purified ^32^P-labeled probe was incubated with 5 μg nuclear extract in a buffered solution (50 mM NaCl, 0.2 mM EDTA, 4% glycerol, 10 mM Tris–HCl) containing 0.05 μg poly(dI–dC) for 30 minutes at room temperature. DNA-protein complexes were separated through a 6% non-denaturing polyacrylamide gel. Gels were analyzed by autoradiography. For competition experiments, NF-κB unlabeled double-stranded consensus oligonucleotide was included in two-fold molar excess over the amount of ^32^P-NF-κB probe. Anti-p65 or anti-p50 were added in some nuclear extracts before electrophoresis.

### CGD patients

Peripheral blood samples were collected from five patients with CGD (Supplementary Table S1) and five healthy individuals (controls) with a written informed consent. Studies using human samples were conducted in accordance with The Ethics Committee on Human Research adopted by The Brazilian Ministry of Healthy and were approved by The Ethical guidelines of Clinics Hospital of Ribeirao Preto Medical School, University of Sao Paulo, Ribeirão Preto, São Paulo State, Brazil (protocol no. 325.272).

### Other assays

Cytokine/chemokine levels were quantified by using enzymatic-linked immunosorbent assay (ELISA) with antibodies from R&D Systems. Lung myeloperoxidase activity was measured as an index of neutrophil sequestration^40^. Plasma concentrations of aspartate-aminotransferase (AST) and urea were measured by using commercial kits (Labtest, Brazil; Bioclin, Brazil). Reduced glutathione (GSH) assays were performed using the GSH-Glo assay kit (Promega) as described by the manufacturer. Cell viability was determined by using ApoScreen^TM^ Annexin V apoptosis Kit (Southern Biotecnology, USA) according to the manufacturer’s instructions.

### Statistics

Analyses were performed using SAS software v9.0 (SAS Institute Inc., USA). The log-rank (Mantel-Cox) test was used to evaluate the survival rates. The Mann-Whitney test was used to assess variables with two experimental groups and the Kruskal-Wallis test followed by Dunn’s post-hoc correction was used for analyses involving more than two groups.

For more details regarding methods, please see Supplementary Information available online.

## Additional Information

**How to cite this article**: Trevelin, S. C. *et al*. Apocynin and Nox2 regulate NF-κB by modifying thioredoxin-1 redox-state. *Sci. Rep.* **6**, 34581; doi: 10.1038/srep34581 (2016).

## Supplementary Material

Supplementary Information
