# Supplementary material for: Apocynin and Nox2 regulate NF-κB by modifying thioredoxin-1 redox-state
Source: Sci Rep. 2016 Oct 4;6:34581. doi: 10.1038/srep34581 (PMC5048297; doi:10.1038/srep34581)
Supplement: Supplementary Information [file srep34581-s1.pdf]

Supplementary information

Apocynin and Nox2 regulate NF-κB by modifying thioredoxin-1 redox-state

Silvia Cellone Trevelin[1,2,3], Célio Xavier dos Santos[3], Raphael Gomes Ferreira[1], Larissa de Sá Lima[2], Rangel Leal Silva[1], Cristoforo Scavone[2], Rui Curi[4], José Carlos Alves-Filho[1], Thiago Mattar Cunha[1], Pérsio Roxo-Júnior[5], Maria-Célia Cervi[5], Francisco Rafael Martins Laurindo[6], John Stephen Hothersall[1], Andrew M Cobb[3], Min Zhang[3], Aleksandar Ivetic[3], Ajay M Shah[3], Lucia Rossetti Lopes[2*†], Fernando Queiroz Cunha[1*†].

Running title: APO and Nox2 regulate NF-κB by modifying TRX-1.

[1]Department of Pharmacology, Ribeirao Preto Medical School, University of Sao Paulo, Ribeirão Preto, Brazil;

[2]Department of Pharmacology, Institute of Biomedical Sciences, University of Sao Paulo, São Paulo, Brazil;

[3]King's College London, British Heart Foundation Centre, Cardiovascular Division, London, United Kingdom;

[4]Department of Biophysics and Physiology, Institute of Biomedical Sciences, University of Sao Paulo, São Paulo, Brazil;

[5]Department of Pediatrics, Ribeirao Preto Medical School, University of Sao Paulo, Ribeirão Preto, Brazil;

[6]Heart Institute, School of Medicine, University of Sao Paulo, São Paulo, Brazil.

*Correspondence to Professor Fernando Queiroz Cunha (fdqcunha@fmrp.usp.br), Department of Pharmacology, Ribeirao Preto Medical School, Bandeirantes Avenue, 3900, 14049-900-Ribeirão Preto, SP, Brazil. Phone: +55 (16) 3315 3223; or Professor Lucia Rossetti Lopes (llopes@usp.br), Department of Pharmacology, Institute of Biomedical Sciences, Lineu Prestes Avenue, 1524, 05508-900-São Paulo, SP, Brazil. Phone: +55 (11) 30917321. [†] These authors contributed equally to this study.

Supplementary Figures 1-12

Supplementary Table S1

Supplementary Methods

SUPPLEMENTARY FIGURES

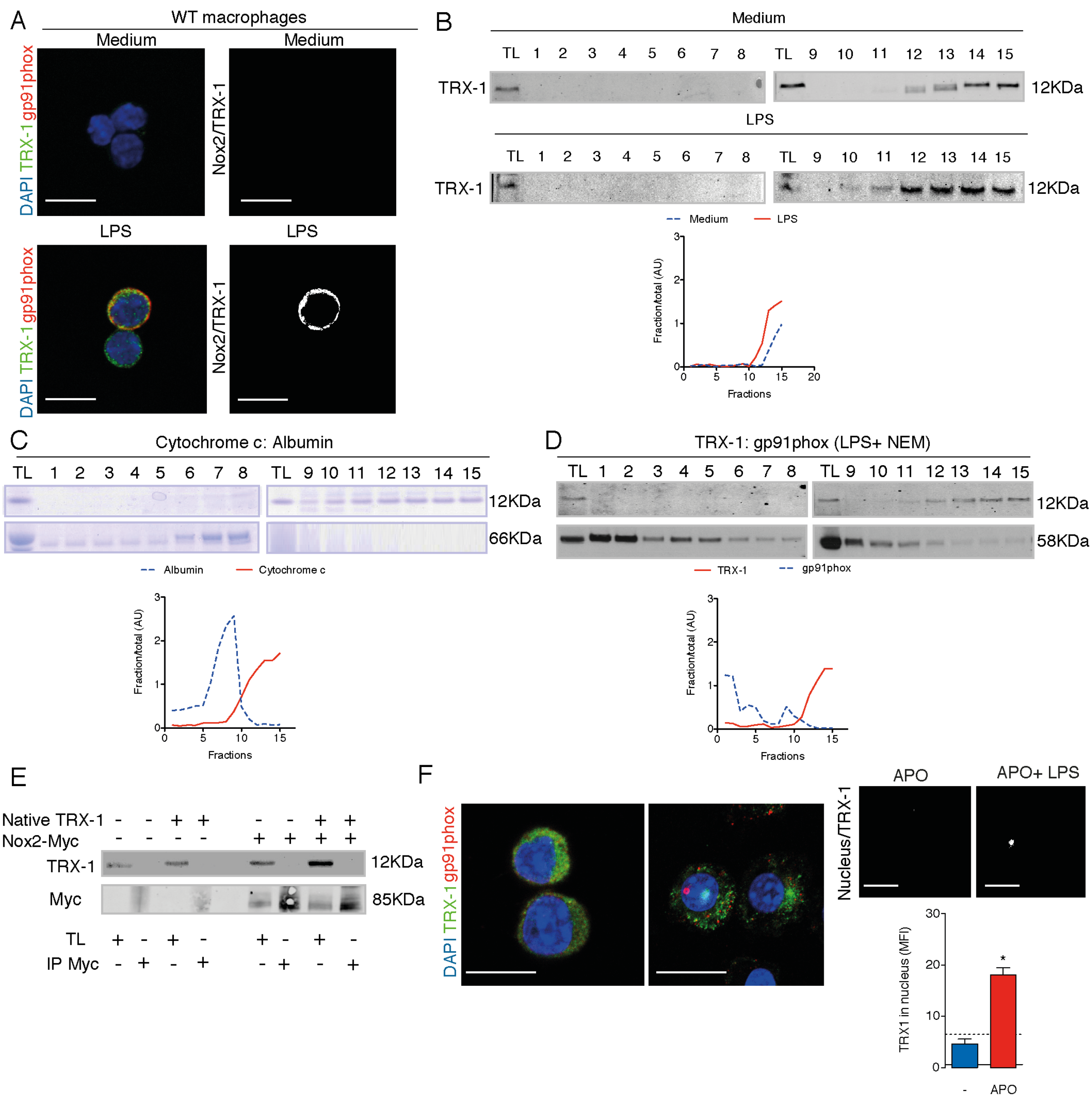

Supplementary Fig. 1. Nox2 activation prevents nuclear accumulation of TRX-1. (A) TRX-1 (green) and gp91phox (red) in peritoneal macrophages harvested from WT mice after LPS stimulation (10 ng/ml, 30 minutes). Nuclear material was stained with DAPI (blue). Images were obtained by confocal microscopy (63X objective; 5X magnification) and represent three independent experiments. Scale bars, 7.5µm. Colocalization is highlighted in white/black image. (B- D) Sucrose gradient cell fractionation and densitometry. (B) Immunoblotting (IB) was performed for TRX-1 in control (medium) or LPS (10 ng/ml, 30 minutes) stimulated RAW264.7 cells. (C) Densitometry for distribution of albumin and cytochrome c (gel filtration molecular weight markers) in individual sucrose gradient fractions, obtained from Commassie blue-stained gels. (D) Lysates of LPS-stimulated RAW264.7 cells were treated with N-ethylmaleimide (NEM, 1mM) 30 minutes before sucrose fractionation. (E) HEK293T cells were transfected with Nox2-Myc and native TRX-1. Immunoprecipitation was performed with anti-Myc antibody (Ab) and IB for Myc and TRX-1. All blots are representative of two independent experiments. TL: total cell lysate. (F) RAW264.7 cells were incubated with apocynin (APO, 300µM, one hour) and stimulated with LPS. Images were obtained by confocal microscopy (63X objective; 5X magnification) and represent one experiment performed in triplicate. Scale bars, 7.5µm. The results are expressed as the means of fluorescence intensity (MFI) ± SEM obtained by analysing 15 nucleus-TRX-1 colocalizations *per* group (white/black images). Black continuous and dashed lines respectively indicate the average values of cells incubated medium or APO-treatment only. *P<0.05 as compared to cells incubated with LPS.

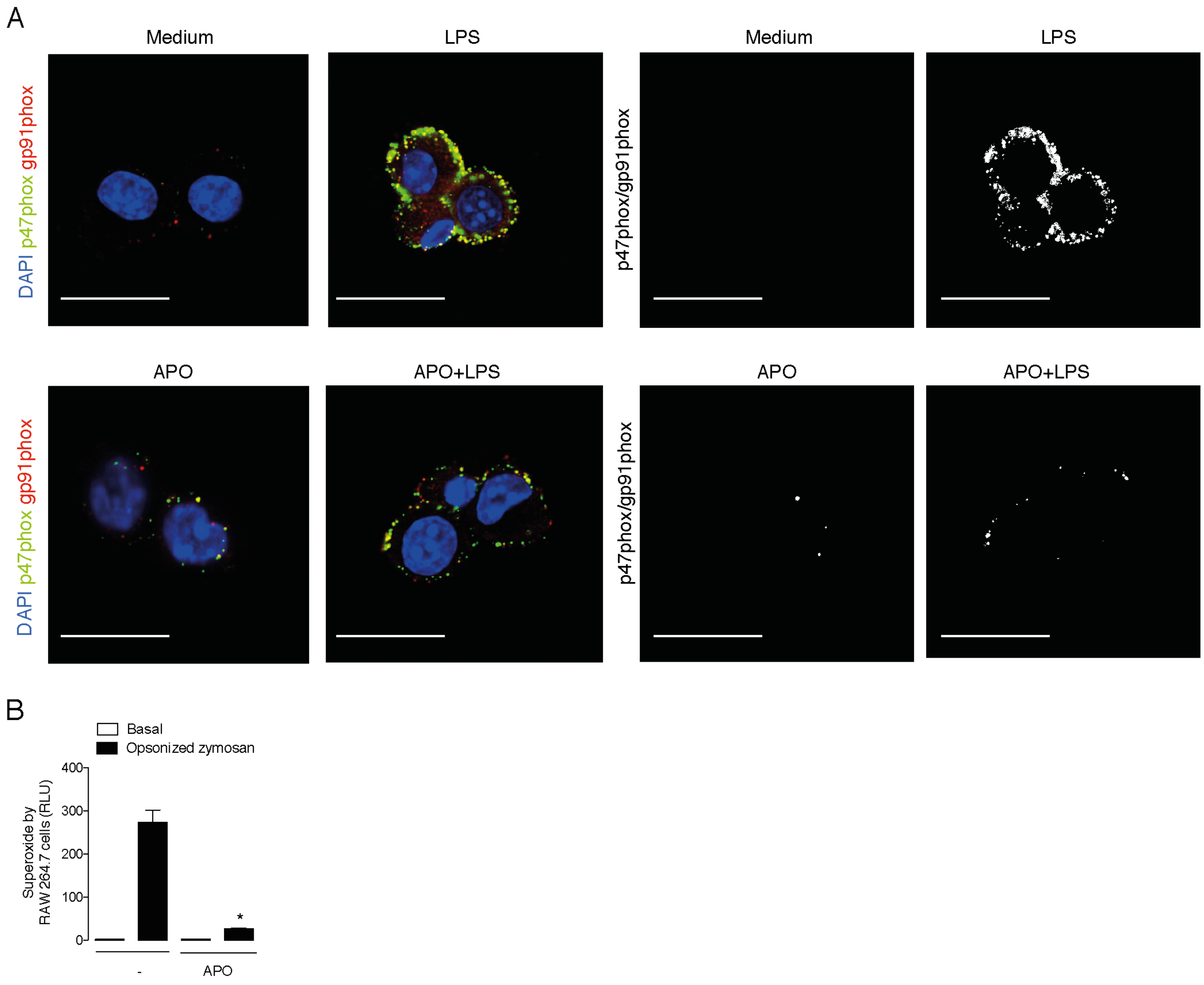

Supplementary Fig. 2. APO impairs Nox2 assembly and reactive oxygen species (ROS) production by leukocytes. (A) RAW264.7 cells were incubated with APO (300 µM, one hour) and then stimulated with LPS (10 ng/ml, 30 minutes). Colocalizations (white/black images) of p47phox (green) and gp91phox (red) were verified using confocal microscopy (63X objective; 5X magnification). Scale bars, 7.5µm. Nuclear material was stained with DAPI (blue). Images are representative of two independent experiments realized in triplet. (B) Superoxide production after stimulation with opsonized zymosan (5 particles/cell). The results are expressed as the means ± SEM (n=3/group). **P*<0.05 as compared to cells stimulated with opsonized zymosan without pre-treatment with APO.

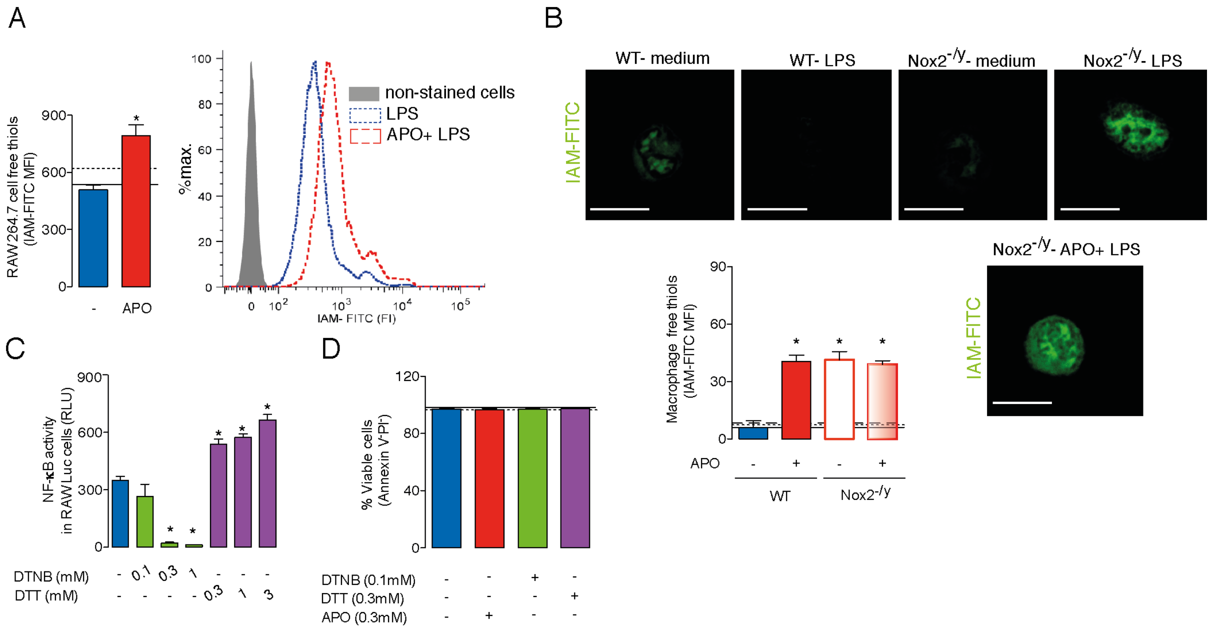

Supplementary Fig. 3. Pharmacological inhibition or genetic deficiency of Nox2 induces a reductive stress, which increases NF-κB activation. RAW264.7 cells (A) or peritoneal macrophages harvested from WT or Nox2$^{-/y}$ mice (B) were incubated with APO (300 µM, one hour) and stimulated with LPS (10 ng/ml, 30 minutes). Levels of free thiols were determined using iodoacetamide conjugated to isothiocyanate fluorescein (IAM-FITC, green). Scale bars, 7.5µm. FI: fluorescence intensity. The results are expressed as the means of fluorescence intensity (MFI) obtained by analyzing 15-20 cells by confocal microscopy or 100,000 events by flow cytometry. (C) RAW264.7-Luc cells were incubated with 5,5'-dithiobis-2-nitrobenzoic acid (DTNB) or dithiothreitol (DTT), one hour before treatment with LPS (10 ng/ml, 4 hours). (D) Cell viability 12 hours after incubation with LPS, APO, DTNB or DTT. Black continuous and dashed lines respectively indicate the average values of cells incubated in medium or APO-treatment only. The reults are expressed as the means ± SEM of three independent experiment realized in triplet. RLU: relative lumen units. *P<0.05 as compared to cells incubated with LPS.

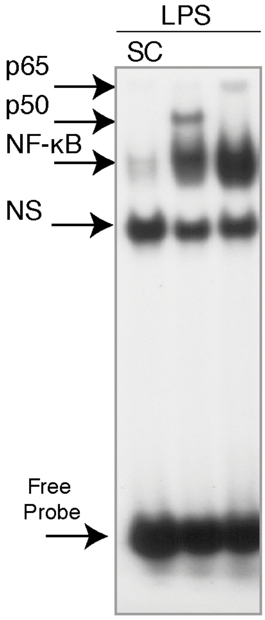

Supplementary Fig. 4. LPS stimulates translocation of the NF-κB subunits p65 and p50 to the nucleus. RAW264.7 cells were stimulated with LPS (10 ng/ml, 30 minutes). Eletrophoretic mobility super-shift assay under reducing (DTT, 1 mM) condition with anti-p50 and anti-p65 antibodies. NF-κB unlabeled consensus oligonucleotide (specific competitor, SC) was included in two-fold molar excess over the amount of radioactive-NF-κB probe in order to detect non-specific DNA- protein interactions. NS: non-specific.

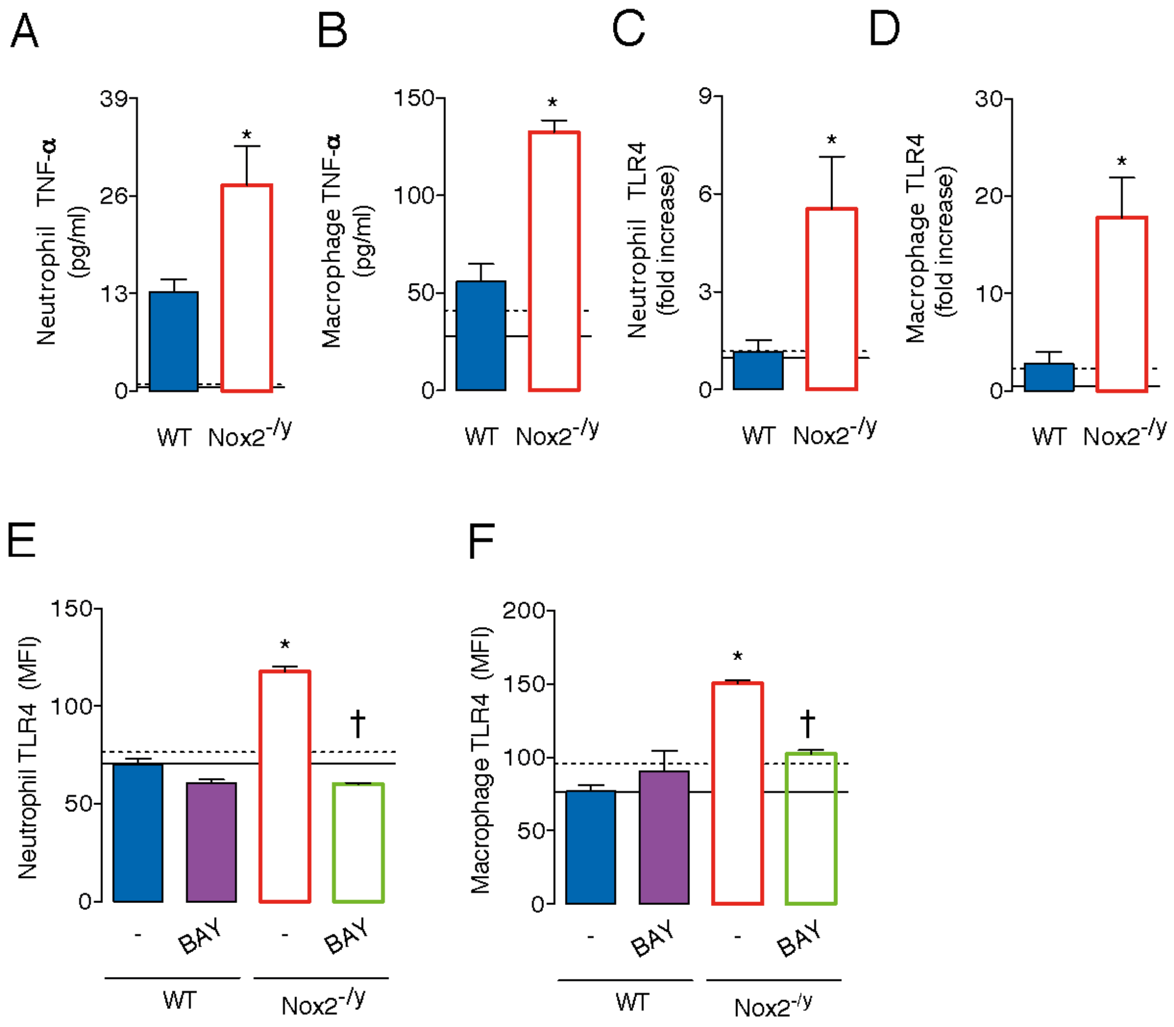

Supplementary Fig. 5. Nox2 deficiency enhances LPS-induced NF-κB transcription of inflammatory molecules. (A-F) Bone marrow (BM) neutrophils or peritoneal macrophages from wild type (WT) or Nox2 deficient ($Nox2^{-/y}$) mice were incubated with LPS (10 ng/ml, six and 12 hours, respectively). (A, B) TNF-α levels in culture supernatant (ELISA); (C,D) TLR4 mRNA (qRT-PCR); (E,F) TLR4 expression assessed by flow cytometry. BAY: BAY 11-7082 (10 µM, 30 minutes before LPS); MIF: median of fluorescence intensity. Black continuous and dashed lines indicate the average values obtained by analysing respectively WT and $Nox2^{-/y}$ cells in medium only. The reults are expressed as the means ± SEM (n=6/group, samples incubated with LPS; n=3/group, samples without LPS). **P*<0.05 as compared to WT cells incubated with LPS only; †*P*<0.05 as compared to $Nox2^{-/y}$ cells incubated with LPS only.

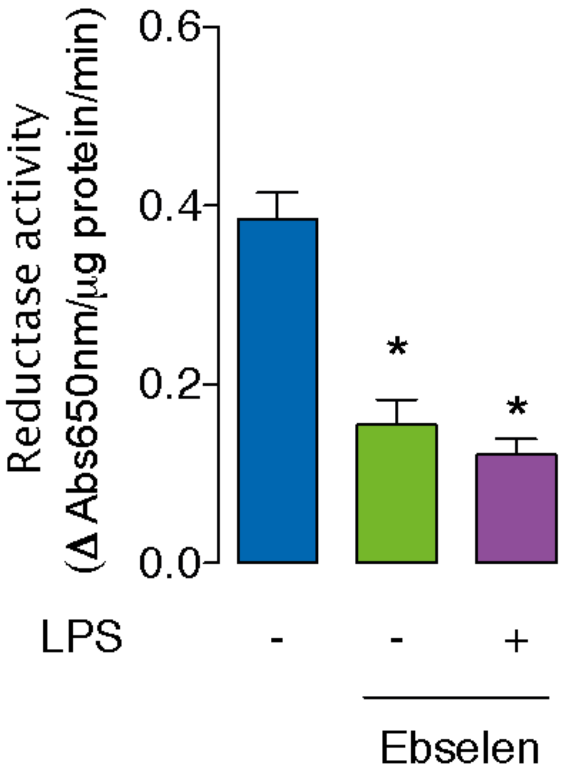

Supplementary Fig. 6. Ebselen impairs TRX-1 mediated reductase activity. RAW264.7 cells were incubated with ebselen (30 μM, one hour) and then stimulated with LPS (10 ng/ml, 30 minutes). The reults are expressed as the means ± SEM (n=3/group). Abs: absorbance. Min: minute. **P*<0.05 as compared to cells in medium only.

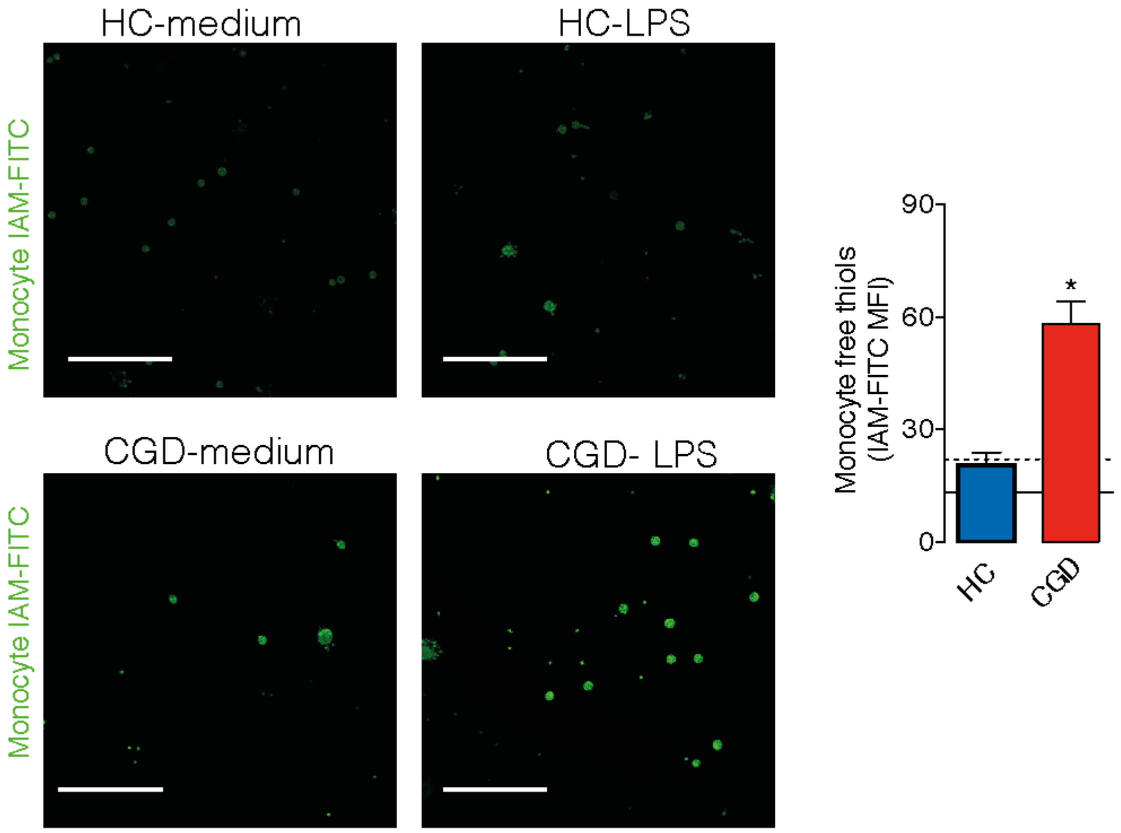

Supplementary Fig. 7. Monocytes from chronic granulomatous disease patients (CGD) exhibit reductive stress. Monocytes from healthy controls (HC, n=5) or CGD patients (n=5) were stimulated with LPS (10 ng/ml, 30 minutes). Images showing cells stained with IAM-FITC (green) were obtained by confocal microscopy (63X objective). Scale bars, 50µm. The results are expressed as the means of fluorescence intensity (MFI) ± SEM obtained by analyzing 15 cells/subject. Black continuous and dashed lines indicate the average values obtained by analysing cells respectively from HC and CGD in medium only. *$P<0.05$ as compared to HC cells incubated with LPS.

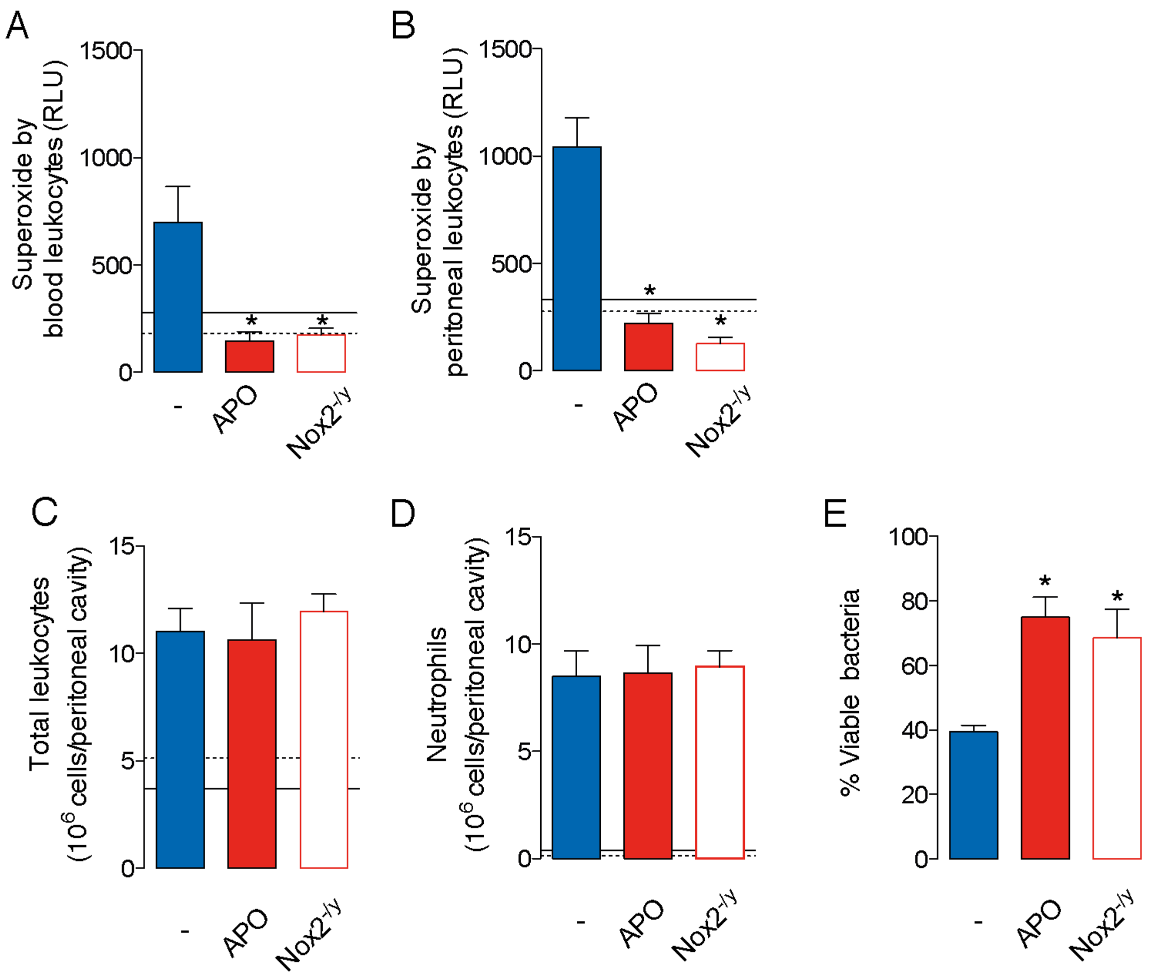

Supplementary Fig. 8. Nox2 activation is required for neutrophil bactericidal activity in sepsis. WT mice subcutaneously treated with APO (200 mg/kg; 30 minutes before surgery) and Nox2$^{-/y}$ mice were submitted to cecal ligation and pucture (CLP). (A, B) Leukocytes obtained from blood or peritoneal lavage were analyzed for superoxide production after stimulation with opsonized zymosan (5 particles/cell). **P*<0.05 compared to WT mice submitted to CLP without pre- treatment with APO. (C and D) Number of total leukocytes and neutrophils harvested from peritoneal cavity six hours after surgery. Black continuous and dashed lines indicate the average values obtained by analysing parameters respectively in WT and WT pre-treated with APO mice submitted to sham surgery. The reults are expressed as the means ± SEM (sham, n=4; CLP, n=5/group). (E) Neutrophils were purified from bone marrow of Nox2$^{-/y}$ or WT mice. WT cells were incubated with APO (300 µM, one hour). Bacterial killing assay with cecal bacteria (n=3/group). **P*<0.05 as compared to WT cells without pre- treatment with APO.

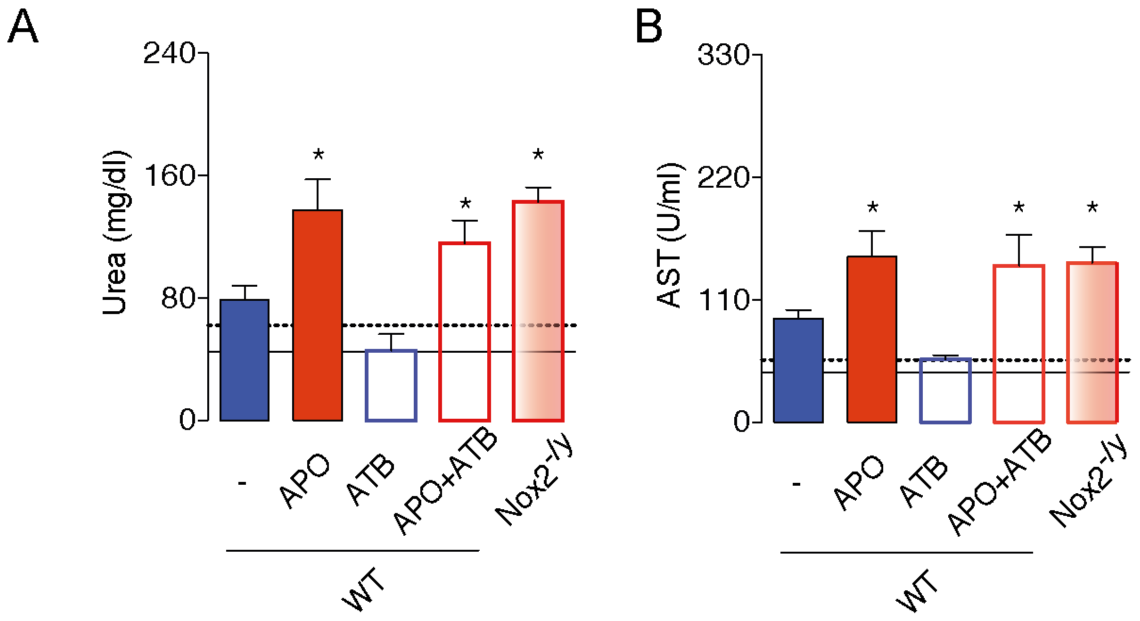

Supplementary Fig. 9. Pharmacological inhibition or genetic deficiency of Nox2 aggravates multi-organ dysfunction in sepsis. WT mice subcutaneously treated with APO (200 mg/kg, 30 minutes before surgery) and Nox2$^{-/y}$ mice were submitted to cecal ligation and puncture (CLP). Some mice were intraperitonously treated with antibiotic (ATB, ertapenem sodium, 30 mg/kg), six hours after surgery and 12 hourly thereafter. Levels of aspartate aminotransferase (AST [A]) and urea (B) in plasma, six hours after ATB treatment and 12 hours after CLP. The results are expressed as the means ± SEM (n=5-7/group). Black continuous and dashed lines indicate the average values obtained by analysing WT and Nox2$^{-/y}$ sham-operated mice, respectively. *P<0.05 as compared to WT-CLP given mice.

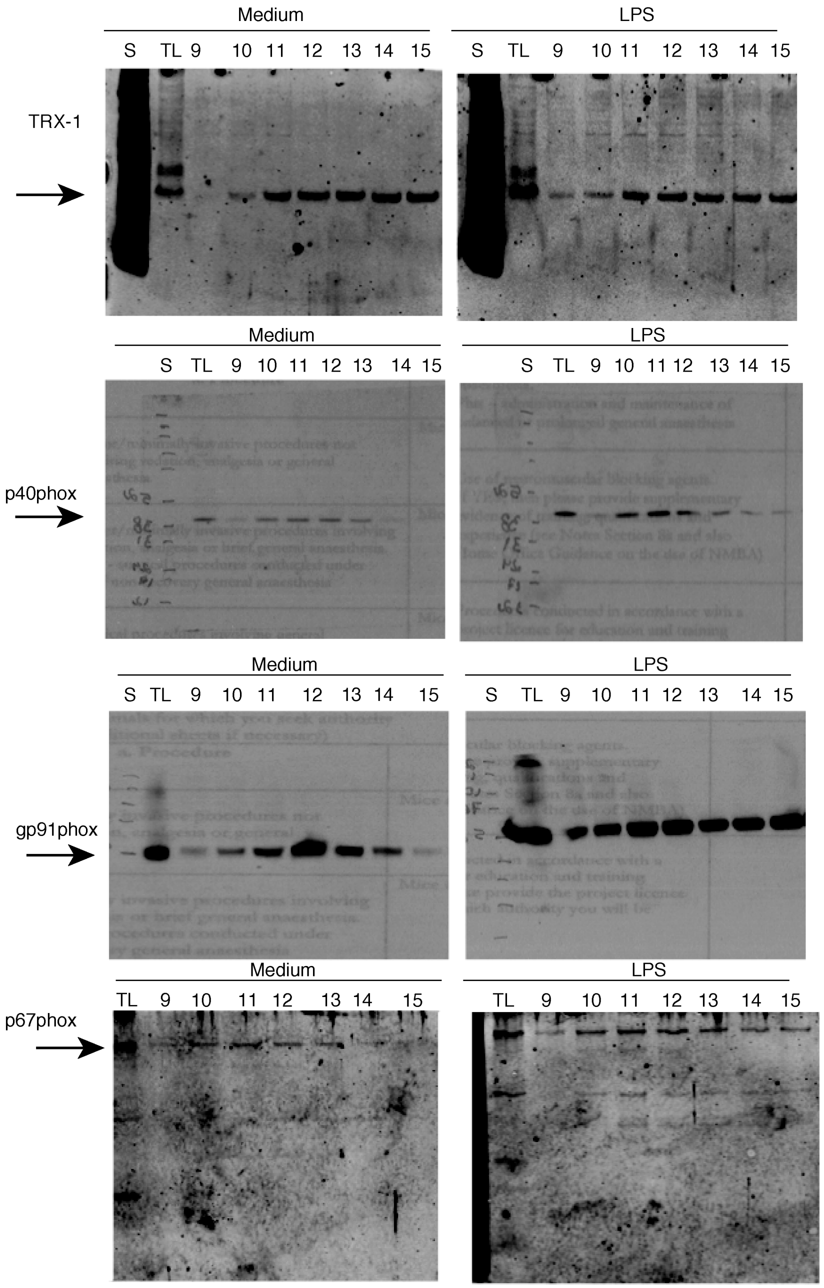

Supplementary Fig. 10. Full-lengh blots of Figure 1B. S: standard molecular weight markers.

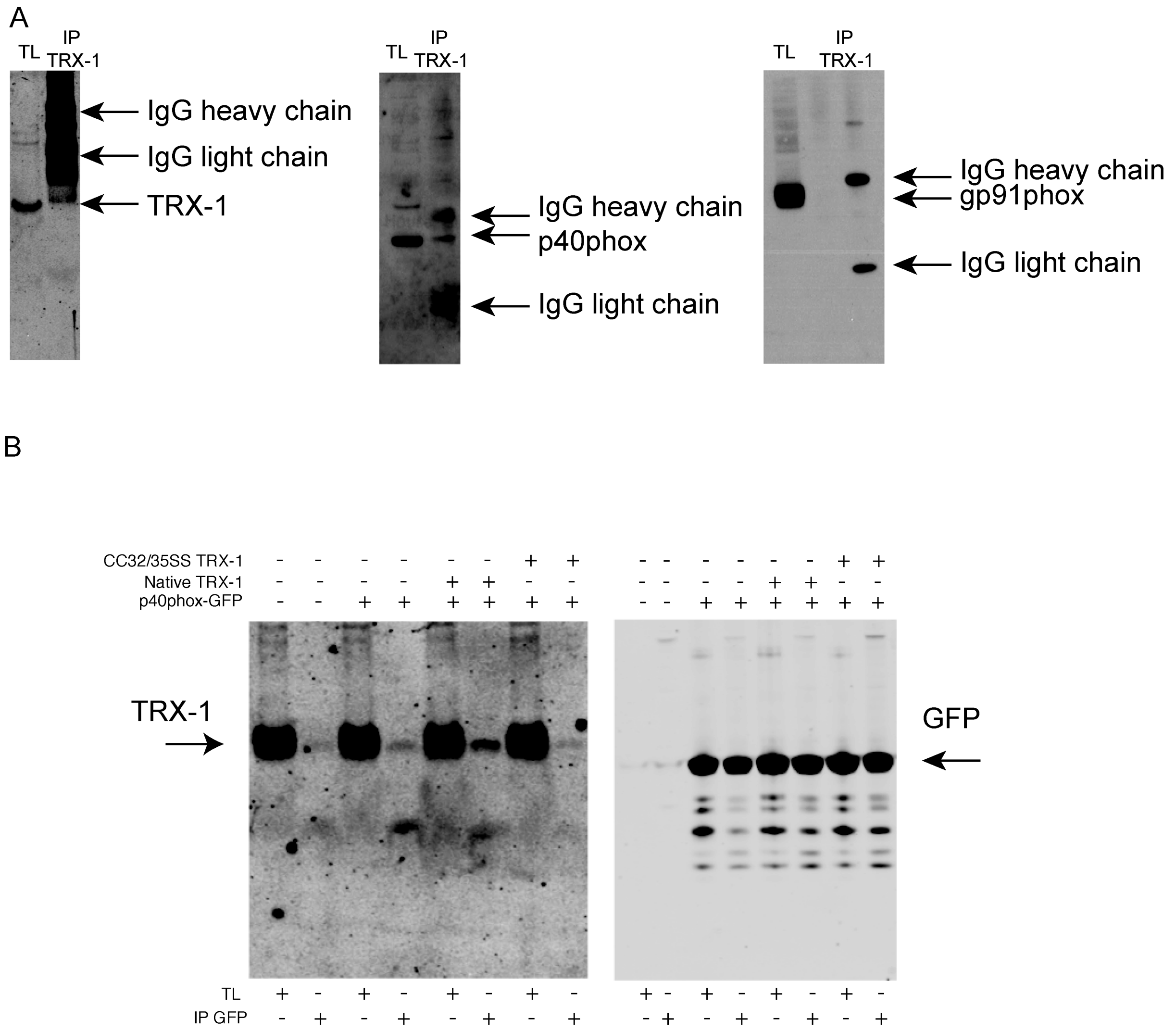

Supplementary Fig. 11. Full-lengh blots of Figures 1C (A) and 1D (B).

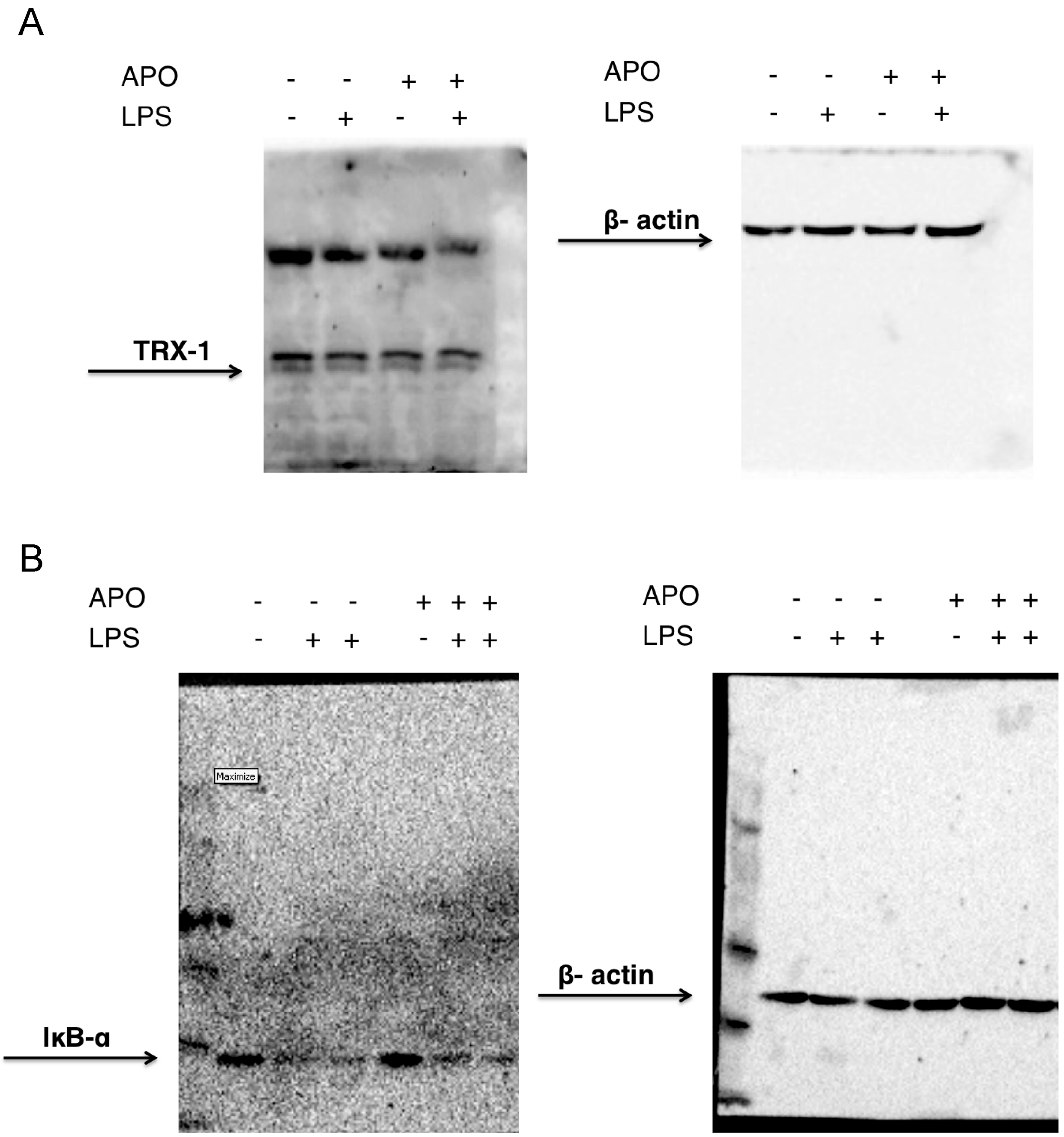

Supplementary Fig. 12. Full-lengh blots of Figures 2B (A) and 3A (B).

SUPPLEMENTARY TABLE S1

Supplementary Table S1. Clinical profile of chronic granulomatous disease patients (CGD) and healthy controls.

	CGD Patients (n=5)	Healthy Controls (n=5)
**Age (years±SEM)**	12.8±1.43	19.8±1.416
**Gender (females vs males)**	2 vs 3	2 vs 3
**Initial diagnostic**		
Hepatic abscess	1	NA*
Family history	1	NA
Pneumonia†	3	NA
**NBT‡ (%positive neutrophils)**		
Basal (mean±SEM)	1±0.44§	15±2.04
Stimulated with PMA¶ (mean±SEM)	2.8±1.16§	78.6±3.65
**NADPH oxidase 2 subunit**		
Nox2 deficiency	2	NA
p47phox deficiency	3	NA

*NA: not applicable; †two patients presented pulmonary aspergilosis and one patient had gram negative bacterial pneumonia; ‡NBT: nitroblue tetrazolium test; ¶PMA: phorbol 12-myristate 13-acetate (PMA) 0.3μM; §P<0.05 as compared to healthy controls.

SUPPLEMENTARY METHODS

Cells and reagents. LPS serotype O157:H7 from *E. coli* was purchased from Sigma-Aldrich (purified by trichloroacetic extraction). Antibody sources were: anti-mouse Nox2 (BD Biosciences); anti-human Nox2, anti-p65, anti-myc, anti-IκB-α and anti-p67phox (Santa Cruz); anti-TRX-1 (Cell Signaling); anti-p47phox and anti-p40phox (Millipore); phycoerythrin-conjugated anti-TLR4 mAb (eBiosciences). RAW264.7 cells were acquired from Life Technologies. The bacterial plasmids p40phox-GFP, pET16b-TRX (TRX-1 native) and pET16b-TRX CC32/35SS were obtained from Addgene (Cambridge, UK).

Detection of reactive oxygen species (ROS). Superoxide production was determined by chemiluminescence using lucigenin (5μM) in a plate luminometer (Mithras LB 940 Multimode Microplate Reader, Berthold technologies, Calmbbacher, Germany). Superoxide generation was analysed in basal condition and in the presence of opsonized zymosan (5 particles/cell). The results were subtracted from values obtained in the presence of superoxide dismutase (SOD- 300μM).

Whole blood samples (25μl) from chronic granulomatous disease patients (CGD) or healthy controls were mixed with nitroblue tetrazolium (25μl of NBT 1mg/ml, Sigma-Aldrich) and incubated at 37° C for 15 minutes, followed by 15 minutes at room temperature. Phorbol 12-myristate 13-acetate (PMA 0.3μM) was added to the samples immediately before incubation with NBT. The percentage of positive cells (containing formazan deposits, *ie* the product of NBT reduction) was estabilished by counting 100 neutrophils in each slide.

Bacterial killing. Bone marrow (BM) neutrophils (10^6 cells) were incubated with cecal bacteria ($2x10^6$ CFU/ $1x10^6$ cells) for three hours at 37°C. After then, the cells were washed with PBS, lysed with Triton 0,2% and sonicated for two minutes. The samples were next centrifuged 500g for 10 minutes and the supernatants plated on Muller-Hinton agar dishes (Difco Laboratories) that were incubated at 37°C. Colony forming units (CFU) were registered after 18 hours.

qRT-PCR. Total RNA was extracted using Trizol reagent (Invitrogen- Life Technologies, Carlsbad, CA). Samples were treated with RQ1 RNASe-free DNAse (Promega. Wiscosin, USA) to avoid amplification of genomic DNA. The synthesis of cDNA was carried out by reverse transcription (ImpromII™ ReverseTranscriptase, Madison, WI, USA) using 1µg of total RNA. Real time PCR (qPCR) analysis was performed using Power Syber® Green PCR master mix (Applied Biosystems, Warrington, UK) and the reactions were processed in Mastercycler® eppendorf realplex (Eppendorf, Hamburg, Germany). The reactions were carried out in final volume of 20µl containing 10µl of SYBR Green with 50µM of each primer and 2 µl of cDNA. The samples were hold at 95° C for 10min followed by 40 cycles at 95 ° C for 15 seconds and 60 ° C for 30 seconds. The melting curve was constructed by increasing temperatures from 60 ° C to 95°C. Comparative threshold cycle method was used for quantification (fold increase= $2^{-\Delta\Delta ct}$). Primers used were: murine TLR4 sense (5’-CCAAGCCTTTCAGGGAATTAA-3’), murine TLR4 antisense (5’-GCCAGGTTTTGAAGGCAAGT-5’), murine GAPDH sense (5’-GGCAAATTCAACGGCACAGT-3’), murine GAPDH antisense (5’-TCCCCACTGCCTACATACCA -3’). GAPDH mRNA levels were used for normalization.

Flow cytometry analysis. $3x10^5$ BM-neutrophils or peritoneal macrophages were incubated with PE-anti-TLR4 mAb (1:100) and PercP anti–Ly6G mAb (1:200). For quantification of free thiols, RAW264.7 cells were incubated with IAM-FITC for 30 minutes. Cells were fixed in 4% paraformaldehyde and analyzed on a BD Biosciences Canto II flow cytometer and Flowjo software 8.7 (©Stanford University, ©Tree Star, Inc.).
